# Health disparities in Turner Syndrome: UTHealth Turner Syndrome Research Registry

**DOI:** 10.20517/rdodj.2023.02

**Published:** 2023-03-16

**Authors:** Priscille Donate, Michelle Rivera-Davila, Siddharth K. Prakash

**Affiliations:** 1 Division of Endocrinology, Department of Pediatrics, University of Texas Health Science Center at Houston, Houston, TX 77030, United States.; 2 Division of Medical Genetics, Department of Internal Medicine, University of Texas Health Science Center at Houston, Houston, TX 77030, United States.

**Keywords:** Registry, Turner Syndrome, health disparities, questionnaire

## Abstract

**Aim:**

Turner Syndrome (TS) is caused by partial or complete absence of the second sex chromosome in a phenotypic female. TS is associated with recognizable congenital anomalies and chronic health conditions. The principal objective of this study was to evaluate the health-related knowledge and insight of participants.

**Methods:**

In 2015, we founded the UTHealth Turner Syndrome Research Registry for longitudinal follow-up of individuals with TS. Study participants were recruited from UTHealth Houston clinics and the Turner Syndrome Society of the United States. Participants completed a questionnaire about demographics, karyotype, congenital anomalies, health history, frequency of contact with care providers, and knowledge of care providers about TS.

**Results:**

Forty percent of registry participants indicated that they did not know their karyotypes. Knowledge of karyotype, which can predict clinical outcomes in TS, markedly varied by self-reported race and ethnicity but not by age. Participants also reported significant gaps in routine medical and gynecologic care.

**Conclusion:**

We identified knowledge gaps and health disparities that could benefit from improved provider and patient education.

## INTRODUCTION

Turner Syndrome (TS) is one of the most common sex chromosome abnormalities, with a prevalence of 1 in 2000 liveborn phenotypic females^[1]^. Individuals with TS may come to clinical attention due to distinctive physical characteristics including short stature, neck webbing, and characteristic chest and limb deformities. TS karyotypes are highly variable and frequently include two or more mosaic aneuploid cell lines. The most common TS karyotype is constitutional monosomy of the entire X chromosome. X chromosome structural variants or Y chromosome fragments may also be present^[2]^.

TS affects many organs and is associated with autoimmune, endocrine, and skeletal disorders. The most common congenital anomalies are cardiovascular and renal malformations. Congenital and acquired cardiovascular diseases are the leading causes of mortality^[3]^. Reproductive health is important to women with TS because most develop primary ovarian insufficiency. If their uterus is present, they also remain at risk for cervical or endometrial cancer and require routine health screening as well as testing for sexually transmitted infections, just as in the general population^[3]^. The complexities of the conditions associated with TS require lifelong monitoring, not only by primary care providers but also by the physician subspecialists that participate in their care.

The University of Texas Health Science Center at Houston (UTHealth Houston) Turner Syndrome Research Registry (NCT03185702) was created to investigate risk factors and long-term outcomes of TS-related health conditions. Participants were recruited from the UTHealth Houston Turner Syndrome Adult Comprehensive Care Center and from individuals who were referred to us by the Turner Syndrome Society of the United States (TSSUS). One objective of the registry is to determine the frequency of congenital anomalies and health conditions in TS. Registry enrollment involves providing a DNA sample (blood, saliva and/or urine) and answering an extensive questionnaire. Participants also provide consent to include their medical record data in the registry. Registry data that is securely stored in a REDCap database includes demographics, karyotypes, clinical findings, laboratory studies, imaging, and clinical diagnoses. Our goal is to investigate genotype-phenotype associations in TS and prioritize gaps in healthcare in the TS community.

The primary objective of this study was to evaluate participants’ self-reported questionnaire data about their TS-related health experiences. The questionnaire was intended to provide an overview of TS-related healthcare in comparison with current clinical practice guidelines, as well as to evaluate the knowledge and insight of participants about their clinical status and healthcare needs. The data may be useful to evaluate how communication of health information between providers and TS patients can be improved.

## METHODS

The study protocol (HSC-MS-15-0120) was reviewed and approved by the Committee to Protect Human Subjects at UTHealth Houston.Registry participants consented to be recontacted for new research studies.Participants or parents (if the participant is under 18 years of age) complete the questionnaire once at study enrollment. The thirty-seven survey items (Supporting Information) include demographics, karyotypes, congenital anomalies, medical diagnoses, access to subspecialists, provider knowledge about TS, and patient satisfaction. Self-reported questionnaire data were extracted from REDCap in May 2021. Demographic data and karyotypes were confirmed by reviewing available medical records. Descriptive variables were compared using chi-squared tests. Quantitative variables were compared using *t*-tests or Fisher exact tests.

## RESULTS

**Figure 1.**
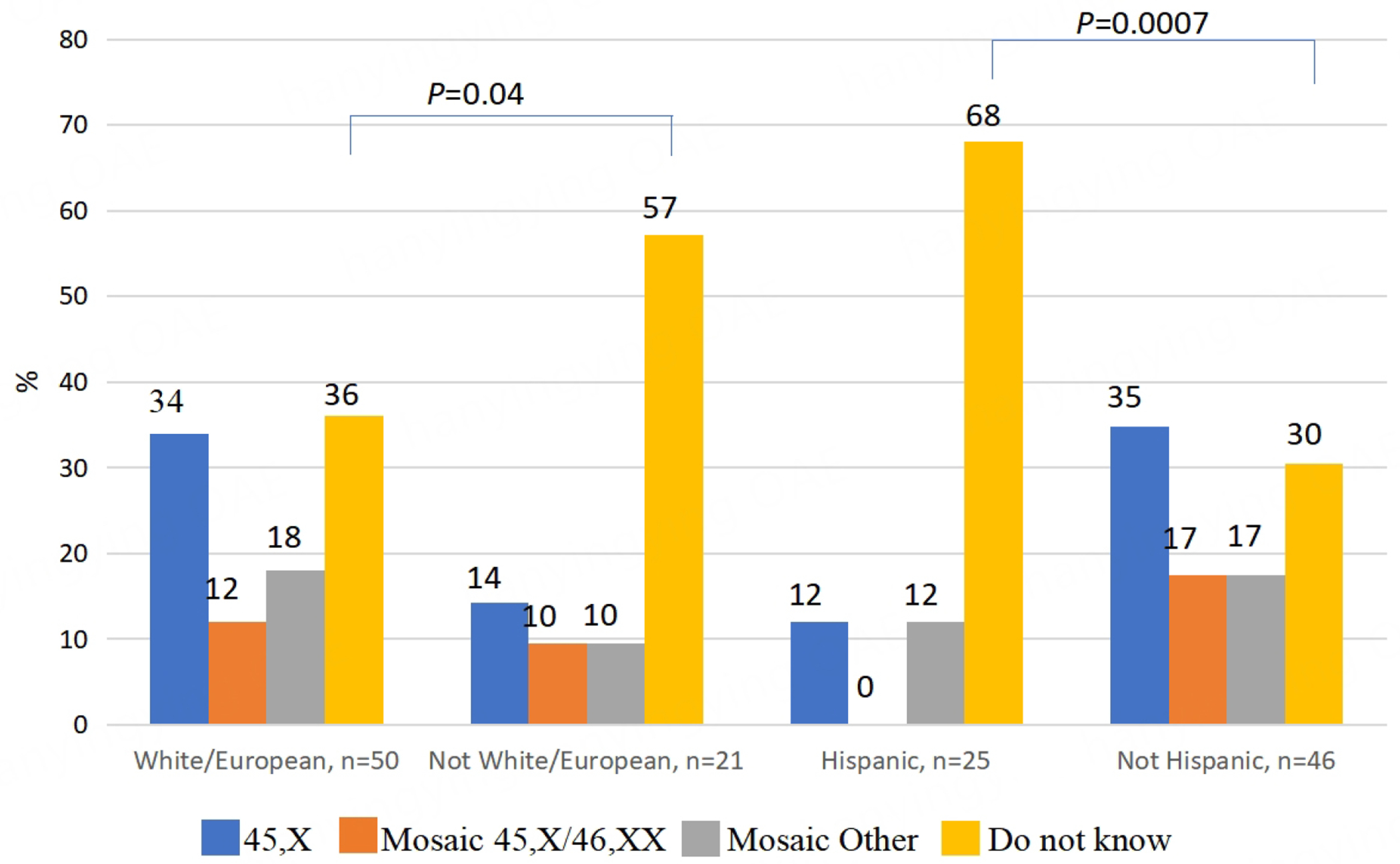
Bar graphs illustrate the percentages of self-reported karyotypes in the UTHealth Houston TS registry cohort by race and ethnicity. There were 25 participants of Hispanic ethnicity and 21 non-White/European participants. Compared to White/European registry participants, significantly more non-White and Hispanic participants did not know their karyotypes. Age was not significantly associated with knowledge of karyotype (*P* = 0.9).

There were 88 total registry participants. Complete questionnaires from 72 individuals with TS (55 adults and 17 children) were selected for review [Table 1]. Sixteen surveys were excluded because they were not entirely completed. The mean age of adult participants was 35 years and the mean age of child participants was 11 years. The most common self-reported karyotypes were 45,X or 45X/46,XX. Strikingly, 40% (29/72) of registry participants indicated that they did not know their karyotypes. Knowledge of karyotype markedly varied by self-reported race and ethnicity [Figure 1]. More than two-thirds of Hispanic participants and 57% of non-White participants were unaware of their karyotypes, as compared to 17% of participants who identified as not Hispanic and White/European (*P* = 0.0007, Figure 1). Knowledge of karyotype was not correlated with the education level of participants.

Congenital heart lesions, principally bicuspid aortic valve (BAV), coarctation, and thoracic aortic disease, were diagnosed in half of the study participants. While all registry participants had echocardiograms, only 24 (33%) self-reported a CHD diagnosis. The relatively small cohort of Black and Asian participants did not report any CHD lesions. There were no significant racial or ethnic differences in the proportion of participants who had been evaluated by a cardiologist within the last 10 years.

Reproductive healthcare was variable in the registry cohort. Half of the adult registry participants reported that they had a pelvic ultrasound. Ultrasound is clinically indicated in adult patients with TS to assess the uterus and is therefore a benchmark for quality of care in TS^[3]^. Absence of the uterus or uterine anomalies raises concern for gonadoblastoma^[4]^. Approximately equal proportions indicated that they were found to have a healthy uterus and/or normal ovaries (34%) or a small uterus and/or streak ovaries (30%). Surprisingly, 20% of adult participants (11/55) reported that they had never seen a gynecologist. This proportion was higher if they considered their primary care provider (PCP) to be a pediatrician (66%) or did not have a regular primary care provider (57%).

TS is usually accompanied by hypergonadotropic hypogonadism and primary or secondary amenorrhea due to gonadal dysgenesis. Most women with TS require hormone replacement therapy (HRT)^[3]^. We found that only 40% of the adult registry participants endorsed a history of ovarian failure. In 80% of these cases, HRT was required to induce breast development or menses. Thirty percent of study participants did not recall if they had ever received HRT.

The spectrum of renal anomalies in patients with TS includes horseshoe kidney (10%), ureteral duplication (5%–10%) and renal agenesis (3%). A renal ultrasound is therefore recommended at diagnosis. In the UTHealth Houston cohort, more than one-third of study participants (36%) were not aware of their renal status. The rate of self-reported renal structural abnormalities among participants who knew their imaging results was 66%, including 22% with horseshoe kidneys.

Hearing impairment is prevalent in TS due to craniofacial structural abnormalities and sensorineural or conductive hearing loss. In the UTHealth Houston cohort, 27 participants (38%) had hearing impairment and received regular follow-up visits with an audiologist. However, most participants who did not endorse hearing loss (25/45) had not been evaluated by an audiologist at the time of the study.

Self-reported anxiety (60%) and depression (75%) were prevalent in the study cohort. The most frequent neurodevelopmental issues were Attention Deficit Hyperactivity Disorder (ADHD) or learning disabilities (25%). The most difficult school subject for participants was mathematics.

Participants were asked to rate the comfort or insight of their primary care provider (PCP) into TS-related topics. Most participants agreed that their PCP demonstrated a solid understanding of TS, although this varied by provider type and was lowest among those whose PCP was not an internist or pediatrician. Most participants also indicated that they were able to discuss challenging or difficult health topics with their PCP, regardless of their specialty.

## DISCUSSION

Health disparities are differences in health services or health outcomes that are caused by systemic racial, social, economic, or environmental factors^[5]^. Our data indicate that limited access to care may drive significant health disparities in core measures of healthcare in TS. The UTHealth Houston tertiary clinic registry cohort generally adheres to guideline-recommended surveillance care for TS-related conditions. Most participants had seen a cardiologist within 10 years and had at least one pelvic sonogram or audiology evaluation. However, we identified pronounced disparities in knowledge and insight about TS among participants who identify as Hispanic or Black. Hispanic or Black participants were less likely to know their karyotypes, have regular contact with medical professionals or receive guideline-recommended follow-up visits for TS-related conditions. Individuals who knew their specific karyotype were predominately White and were more likely to report routine follow-up by a primary care physician. Disparities in access to regular care are especially important in TS, which requires nuanced lifelong multisystem surveillance and treatment. Black and Hispanic participants are notably underrepresented in contemporary TS clinical studies, so there is little data about the impact of race on TS care and outcomes^[5–8]^. However, undertreatment of non-White participants does affect outcomes in other chronic health conditions^[9–12]^.

We found that the clinical characteristics of registry participants largely represented published data, with one notable exception being the prevalence of mental health disorders. Anxiety, depression, attention issues, and learning difficulties were prevalent in the UTHealth Houston registry cohort. Math learning difficulties are frequent in TS and are most likely related to visuospatial learning deficits and dyscalculia^[3]^. The prevalence and severity of mood disorders in people with TS are unclear due to the complex interactions between mood, neuropsychological impairment, and chronic illness. Mental illness may affect health outcomes in TS by altering how people interact with care providers or adhere to treatment^[9]^. These data indicate that assessing and treating mental health should be prioritized in future studies.

Another area of concern was limited participation in reproductive health maintenance visits. Pelvic ultrasounds and gynecological exams are essential to detect uterine abnormalities and to screen for gonadoblastoma, a germ cell tumor, in people with TS. Study participants who had not seen a gynecologist may therefore be at increased risk for cancer as well as sexually transmitted infections. In response to these findings, we plan to increase referrals to reproductive health services at future clinical encounters. Additional research is needed to understand why relatively few women with TS receive routine gynecologic care.

The results of this study indicate that a targeted approach aimed at increasing awareness about key features of TS and the importance of regular clinical surveillance in Black and Hispanic communities may reduce health disparities. Social media campaigns to improve health knowledge and promote self-care can be an effective channel to reach underrepresented communities^[13–15]^. For TS, an effective educational campaign may include a list of questions to ask at clinic visits and promulgation of the “transition passport” that was developed so that patients can anticipate their care needs^[16]^. This approach may be more likely to succeed if the educational materials are made available in Spanish and are paired with outreach to primary care providers that reinforce guideline-directed care of patients with TS^[13]^. We plan to utilize these strategies in future clinical encounters. A nationwide collaborative network of Turner syndrome clinics, including the Turner Syndrome Adult Comprehensive Care Center at UTHealth Houston, is well positioned to lead these efforts.

### Limitations:

Patient-reported data is subject to recall bias. Subgroup analysis of non-White participants is not possible because they were significantly underrepresented in our survey.

## Supplementary Material

Supplementary Data

## Figures and Tables

**Table 1.** Characteristics of UTHealth Houston Turner Syndrome Research Registry participants (*n* = 72)

Age less than 18 years	17 (23)
White/European	50 (69)
Hispanic	25 (34)
High school or GED	23 (32)
College or Post-graduate	30 (42)
**Reason for diagnosis**	
Prenatal	8 (11)
Short stature	37 (51)
Other physical features	19 (26)
Pubertal delay	21 (29)
Congenital heart lesions	6 (8)
Menstrual or Fertility	17 (23)
**Medical history**	
Diabetes mellitus	5 (7)
Thyroid disease	22 (31)
Hyperlipidemia	11 (15)
Celiac disease	1 (2)
Elevated liver enzymes	8 (11)
Obesity	13 (18)
Osteopenia or osteoporosis	22 (31)
Primary ovarian insufficiency	22 (31)
Estrogen for pubertal induction	19 (26)
Estrogen for primary amenorrhea	16 (22)

Results are shown as *n* (percentage). The number of diagnoses exceeds the number of participants because some participants endorsed more than one diagnosis.

## Data Availability

Not applicable.
