# Supplementary material for: Health disparities in Turner Syndrome: UTHealth Turner Syndrome Research Registry
Source: Rare Dis Orphan Drug J. Author manuscript; Available in PMC 2023 Aug 3. (PMC10398636; doi:10.20517/rdodj.2023.02)
Supplement: Supplementary Data [file NIHMS1914729-supplement-Supplementary_Data.docx]

Health Disparities in Turner Syndrome: UTHealth Turner Syndrome Research Registry

Priscille Donate^1^, Michelle Rivera-Davila^1^, Siddharth K Prakash^2^

**Supplementary Material: Questionnaire**

1. What do you consider yourself to be?
2. White/Caucasian
3. Black/ African American
4. American Indian
5. Native Hawaiian/Pacific Islander
6. Alaska Native
7. Asian
8. Other
9. Ethnicity?
10. Hispanic
11. Non-Hispanic
12. What is your highest level of Education?
13. Younger than High school
14. Did not finish high school
15. High School diploma
16. Trade School
17. Some College
18. College Graduate
19. Graduate School
20. Under what circumstances were you diagnosed with Turner Syndrome? (Can check more than one)
21. Diagnosed before birth/prenatal ultrasound
22. Diagnosed based on physical features (puffy hand and feet, wide neck, etc)
23. Diagnosed based on short stature
24. Diagnosed based on lack of puberty
25. Diagnosed based on heart disease
26. Diagnosed based on menstrual irregularities
27. Diagnosed based on fertility problems
28. Other reason
29. Don’t know
30. What is your Karyotype?
31. 45,X
32. 45,X/46,XX
33. 45,X/46,XY
34. Deletion Xp
35. Isochromosome
36. Mosaic with ring chromosome
37. Mosaic with 47,XXX
38. Other mosaic with Y chromosome material
39. Something else
40. Don’t know
41. Have you ever had an Echocardiogram (Ultrasound of the heart?
42. Yes
43. No
44. When was your last Echocardiogram done?
45. Within the past year
46. Less than 5 years ago
47. 5-10 years go
48. More than 10 years ago
49. Don’t know
50. Have you ever had an MRI of the heart?
51. Yes
52. No
53. When was your last cardiac MRI?
54. Within the past year
55. Less than 5 years ago
56. 5-10 years go
57. More than 10 years ago
58. Were you born with a heart defect (congenital heart disease)?
59. Yes
60. No
61. What type of heart defect were you born with? (You can check more than one)
62. Bicuspid aortic valve
63. Coarctation of the aorta
64. Ventricular septal defect
65. Atrial septal defect
66. Abnormal blood vessels going to the heart (long or tortuous)
67. Dilated aorta
68. Other type of defect
69. Don’t know
70. Have you had a bone mineral density test?
71. Yes
72. No
73. Do you have low bone mineral density?
74. Yes
75. No
76. Don’t know
77. What treatments have you received for low bone mineral density?
78. Vitamin D
79. Calcium supplementation
80. Bisphosphonates (like Fosamax)
81. Other treatment
82. None of the above
83. Have you had a renal (kidney) ultrasound?
84. Yes
85. No
86. Do you have any structural kidney abnormalities? (Check all that apply)
87. Horseshoe kidney
88. Small or atrophic kidney
89. Large kidney
90. Problems with the ureters (tubes that bring urine into the bladder)
91. Missing a kidney
92. Cust of the kidney
93. Normal kidneys
94. Other abnormality
95. None of the above
96. Don’t know
97. Have you ever had a pelvic ultrasound?

a. Yes

b. No

1. Did they find…
2. Healthy uterus
3. Healthy ovaries
4. Small uterus
5. Streaked ovaries
6. Something else
7. Don’t know
8. Do you have any of the following medical conditions? (Check all that apply)
9. Diabetes, Type 1
10. Diabetes, Type 2
11. Hypothyroidism
12. Hyperthyroidism
13. Hyperlipidemia (high cholesterol or triglycerides)
14. Celiac Disease
15. Fatty liver
16. Elevated liver enzymes
17. Obesity
18. Sleep apnea
19. None of the above
20. When was your last vision screen?
21. Never
22. More than 10 years ago
23. 5-10 years ago
24. Less than 5 years ago
25. Within the past year
26. When was your last hearing screen?
27. Never
28. More than 10 years ago
29. 5-10 years ago
30. Less than 5 years ago
31. Within the past year
32. Do you have hearing impairment?
33. Yes
34. No
35. Have you suffered from mental illness?
36. Yes
37. No
38. Have you every been diagnosed with any of the following? (Check all that apply)
39. Depression
40. Anxiety
41. Bipolar disorder
42. Attention deficit and hyperactivity disorder (ADD or ADHD)
43. Autisms
44. Developmental delay
45. Learning disability
46. Other
47. None of the above
48. What is/was your best subject in school?
49. Open answer
50. What is/was your worst subject in school?
51. Open answer
52. Wat type of doctor do you consider to be your primary physician?
53. Pediatrician
54. Internist
55. Family practitioner
56. Pediatric endocrinologist
57. Adult endocrinologist
58. Ob-Gyn
59. Cardiologist
60. Nephrologist
61. Another doctor
62. Does your primary doctor seem to understand Turner Syndrome?
63. Yes
64. No
65. Did you feel your doctor did a good job of discussing difficult topic, such as fertility? Why or why not?
66. Open answer
67. Endocrinologist
68. Never
69. More than 10 years
70. 5-10 years ago
71. Less than 5 years ago
72. Within the past year
73. Not sure
74. Cardiologist (Heart specialist)
75. Never
76. More than 10 years
77. 5-10 years ago
78. Less than 5 years ago
79. Within the past year
80. Not sure
81. Gynecologist
82. Never
83. More than 10 years
84. 5-10 years ago
85. Less than 5 years ago
86. Within the past year
87. Not sure
88. Audiologist (Hearing doctor)
89. Never
90. More than 10 years
91. 5-10 years ago
92. Less than 5 years ago
93. Within the past year
94. Not sure
95. Psychologist, Psychiatrist or Counselor
    a. Never
96. More than 10 years
97. 5-10 years ago
98. Less than 5 years ago
99. Within the past year
100. Not sure
101. Do you have ovarian failure?
102. Yes
103. No
104. Don’t know
105. Did you require hormone replacement with estrogen to have breast development?
106. Yes
107. No
108. Did you/Do you require hormone replacement therapy to maintain menstruation?
109. Yes
110. No
